# Multipole expansion of integral powers of cosine theta

**DOI:** 10.1038/s41598-020-77234-4

**Published:** 2020-11-18

**Authors:** E. O. Jobunga, O. S. Okeyo

**Affiliations:** 1 Department of Mathematics and Physics, Technical University of Mombasa, P. O. Box 90420-80100, Mombasa, Kenya; 2 Department of Physics and Materials Science, Maseno University, Private Bag-40105, Maseno, Kenya

**Keywords:** Physics, Atomic and molecular physics, Electronic structure of atoms and molecules

## Abstract

Legendre polynomials form the basis for multipole expansion of spatially varying functions. The technique allows for decomposition of the function into two separate parts with one depending on the radial coordinates only and the other depending on the angular variables. In this work, the angular function $\cos ^k \theta$ is expanded in the Legendre polynomial basis and the algorithm for determining the corresponding coefficients of the Legendre polynomials is generated. This expansion together with the algorithm can be generalized to any case in which a dot product of any two vectors appears. Two alternative multipole expansions for the electron–electron Coulomb repulsion term are obtained. It is shown that the conventional multipole expansion of the Coulomb repulsion term is a special case for one of the expansions generated in this work.

## Introduction

The function $x^k$, where $x=\cos \theta$ and *k* is an integer, is generated in any power series expansion involving a dot product of any two vectors with $\theta$ as an angle between them. The Taylor expansion of the plane wave $\displaystyle e^{i\, {\mathbf {q}} \cdot {\mathbf {r}}}$ (where ${\mathbf {q}}$ is a wave vector of length *q* and ${\mathbf {r}}$ is a position vector of length *r*) and Coulomb repulsion term in many-body systems are two classic examples where the exponential term $x^k$ is present.

The multipole expansion is a powerful mathematical tool useful in decomposing a function whose arguments are three-dimensional spatial coordinates into radial and angular parts. This simplifies the solution of physical problems by reducing the triple integrals into a one-dimensional integral of the radial part and the two angular integrals. The angular integrals are solved using angular momentum algebra  ^1,2^. The multipole expansion involves expressing a function as a linear combination of Legendre polynomials, or the related spherical harmonics, with the orders of expansion in this case being the orders of the poles in the multipole expansion ^3^. Many special functions, such as ordinary and spherical Bessel functions^3–5^, arise naturally as the radial part of a function and spherical harmonics as the angular part whenever a function is separated by the multipole expansion series.

In this work, $x^k$ is expanded as a function of the Legendre polynomials. The pattern formed by a sequence of coefficients of the first few Legendre polynomials is analyzed and consequently a generalization equivalent to literature values^3,6^ is derived. The generalization is used for the multipole expansion of the plane wave $\displaystyle e^{i\, {\mathbf {q}} \cdot {\mathbf {r}}}$ and electron-electron interaction term $\displaystyle \frac{1}{|\, {\mathbf {r}}_i-{\mathbf {r}}_j \,|}$. Two equivalent multipole expansion series for the electron-electron interaction are obtained. The conventional multipole expansion of the electron-electron interaction is found to be a special case for one of the expansion series.

## Theory

The Legendre polynomials $\displaystyle P_l( \cos \theta )$ of order *l*, with *l* a non-negative integer, are smooth functions defined in the region $\displaystyle -1 \le \cos \theta \le +1$. They are usually expressed as a power series,

1 $$\begin{aligned} P_l(x) = \sum _{k=\mathrm {0\; {\mathrm {or}}\, 1}}^l c_k\, x^k , \end{aligned}$$

with real number coefficients $c_k$ and where ${x=\cos \theta }$. The summation in Eq. (1), runs from zero (0) for even values of *l* while for odd values, it runs from one (1). The first two Legendre polynomials are ${P_0(x)=1}$ and ${P_1(x)=x}$. Higher-order Legendre polynomials can be generated using the following recurrence relations^3^

2a $$\begin{aligned} (l+1)\,P_{l+1}(x)&=(2l+1)\,x\,P_l(x)-l\,P_{l-1}(x)\,, \end{aligned}$$

2b $$\begin{aligned} \frac{x^2-1}{l}\frac{\mathrm {d}}{\mathrm {d}x}P_l(x)&= x\,P_l(x)-P_{l-1}(x)\,, \end{aligned}$$

2c $$\begin{aligned} (2l+1)\,P_l(x)&= \frac{\mathrm {d}}{\mathrm {d}x}\left[ P_{l+1}(x)-P_{l-1}(x) \right] \,. \end{aligned}$$

In this work, an expansion is done in a reverse process by expressing $x^k$ as

3 $$\begin{aligned} \displaystyle x^k = \sum _{l=0\; \mathrm or \; 1}^k a_l(k)\, P_l(x) \end{aligned}$$

in the Legendre polynomial basis and then deriving the functional dependence of the computed real number coefficient $a_l(k)$ on orders *l* and *k*. Similar to Eq. (1), the summation runs from zero (0) if *k* is even and from one (1) if *k* is odd. The first ten (10) Legendre polynomials^3^ are expressed in terms of $x^k$, where ${x=\cos \theta }$, in Table 1.

**Table 1:** Legendre polynomials $P_l(x)$^3^.

*l*	$P_l(x)$
0	$\displaystyle 1$
1	$\displaystyle x$
2	$\displaystyle \frac{1}{2} \left( 3x^2-1\right)$
3	$\displaystyle \tfrac{1}{2} \left( 5x^3-3x\right)$
4	$\displaystyle \tfrac{1}{8} \left( 35x^4-30x^2+3\right)$
5	$\displaystyle \tfrac{1}{8} \left( 63x^5-70x^3+15x\right)$
6	$\displaystyle \tfrac{1}{16} \left( 231x^6-315x^4+105x^2-5\right)$
7	$\displaystyle \tfrac{1}{16} \left( 429x^7-693x^5+315x^3-35x\right)$
8	$\displaystyle \tfrac{1}{128} \left( 6435x^8-12012x^6+6930x^4-1260x^2+35\right)$
9	$\displaystyle \tfrac{1}{128} \left( 12155x^9-25740x^7+18018x^5-4620x^3+315x\right)$

In the reverse process, $x^k$ can be expanded by writing it in terms of $P_k(\cos \theta )$ and lower powers of *x*, and then similarly replacing the lower powers of *x* with the coresponding functions of Legendre polynomials. This implies that the reverse process has to begin with $x^0$, $x^1$, up to $x^{k}$, in the ascending order. Beginning from the first two cases, ${x^0 = P_0(x)}$ and ${x^1 = P_1(x)}$, already defined in Table 1, the higher order cases of the reverse process are evaluated recursively as follows:

4 $$\begin{aligned} x^2= & {} \frac{1}{3}(2P_2(x)+1) = \frac{1}{3}P_0(x) + \frac{2}{3}P_2(x), \end{aligned}$$

5 $$\begin{aligned} x^3= & {} \frac{1}{5}(2P_3(x)+3x) = \frac{3}{5}P_1(x) + \frac{2}{5}P_3(x). \end{aligned}$$

In Eqs. (4) and (5), we have substituted for 1 in the expression for $x^2$, and for *x* in the expression for $x^3$, with the corresponding predefined lower order cases, respectively. Following the same recursive process, the next higher order cases $x^4$ and $x^5$ are evaluated as

6 $$\begin{aligned} \displaystyle x^4 =\frac{1}{35}\left( 8P_4(x)+30x^2 -3 \right) =\frac{1}{35}\left( 8P_4(x)+30\left[ \frac{1}{3}P_0(x) + \frac{2}{3}P_2(x)\right] -3P_0(x)\right) = \frac{1}{5}P_0(x) + \frac{4}{7}P_2(x) + \frac{8}{35}P_4(x) \end{aligned}$$

and

7 $$\begin{aligned} \displaystyle x^5 =\frac{1}{63}\left( 8P_5(x)+70x^3-15x\right) =\frac{1}{63}\left( 8P_5(x)+70\left[ \frac{3}{5}P_1(x) + \frac{2}{5}P_3(x)\right] -15 P_1(x)\right) = \frac{3}{7}P_1(x) + \frac{4}{9}P_3(x) + \frac{8}{63}P_5(x) \end{aligned}$$

respectively. Likewise, the expansions of $x^6$, $x^7$, $x^8$, and $x^9$ are obtained as

8 $$\begin{aligned} \displaystyle x^6= & {} \frac{1}{7}P_0(x) + \frac{10}{21}P_2(x)+ \frac{24}{77}P_4(x) + \frac{16}{231}P_6(x), \end{aligned}$$

9 $$\begin{aligned} x^7= & {} \frac{1}{3}P_1(x) + \frac{14}{33}P_3(x)+ \frac{8}{39}P_5(x) + \frac{16}{429}P_7(x), \end{aligned}$$

10 $$\begin{aligned} x^8= & {} \frac{1}{9}P_0(x) + \frac{40}{99}P_2(x)+ \frac{48}{143}P_4(x) + \frac{64}{495}P_6(x) + \frac{128}{6435}P_8(x), \end{aligned}$$

11 $$\begin{aligned} x^9= & {} \frac{3}{11}P_1(x) + \frac{56}{143}P_3(x)+ \frac{16}{65}P_5(x) + \frac{192}{2431}P_7(x) + \frac{128}{12155}P_9(x), \end{aligned}$$

respectively. The computed expansion coefficients for $x^k$, for ${0 \le k \le 9}$, with even and odd powers are listed in Table 2.

## Results

Our goal in this study is to obtain a multipole expansion of $x^k$, with order *k* being a non-negative integer and where ${x= \cos \theta }$ is generated from a dot product of two vectors. The natural basis functions for the multipole expansion are the Legendre polynomials, or spherical harmonics which have correspondence relation with the Legendre polynomials. In this work, we have chosen the Legendre polynomials as the basis functions.

Table 2 shows the coefficients, $a_l(k)$, of the Legendre polynomials, $P_l(x)$, in the basis expansion of $x^k$ computed using Eq. (4) up to Eq. (11) for the even and odd values of *k* and *l* respectively.

**Table 2:** Sequence of coefficients, $a_l(k)$, of the Legendre polynomials, $P_l(x)$, computed for the even and odd values of *l* and *k* in the basis expansion of $x^k$ given by Eq. (4) up to Eq. (11).

$x^k/a_l(k)$	$l=0$	$l=2$	$l=4$	$l=6$	$l=8$	$x^k/a_l(k)$	$l=1$	$l=3$	$l=5$	$l=7$	$l=9$
$x^0$	1	–	–	–	–	$x^1$	1	–	–	–	–
$x^2$	1/3	2/3	–	–	–	$x^3$	3/5	2/5	–	–	–
$x^4$	1/5	4/7	8/35	–	–	$x^5$	3/7	4/9	8/63	–	–
$x^6$	1/7	10/21	24/77	16/231	–	$x^7$	3/9	14/33	8/39	16/429	–
$x^8$	1/9	40/99	48/143	64/495	128/6435	$x^9$	3/11	56/143	16/65	192/2431	128/12155

The next task involves forming the sequence of coefficients corresponding to each order of the Legendre polynomial and deductively determining the pattern with the considered cases. Noting that ${k = l, l+2, \cdots }$, we express the sequences for $a_l(k)$ with the corresponding algebraically deduced parametric dependence on *k* for each sequence as:

12 $$\begin{aligned} a_0(k):&\, \displaystyle&1 ,\quad \frac{1}{3} ,\quad \frac{1}{5} , \quad \frac{1}{7} ,\quad \frac{1}{9} ,\quad \cdots ,\quad \frac{1}{k+1} \end{aligned}$$

13 $$\begin{aligned} a_1(k):&\, \displaystyle&\frac{3}{3} ,\quad \frac{3}{5} ,\quad \frac{3}{7} ,\quad \frac{3}{9} ,\quad \frac{3}{11} , \quad \cdots ,\quad \frac{3}{k+2} \end{aligned}$$

14 $$\begin{aligned} a_2(k):&\, \displaystyle&\frac{2}{3} ,\quad \frac{4}{7} ,\quad \frac{10}{21} ,\quad \frac{40}{99} ,\quad \cdots , \quad \frac{k \times 5}{(k+1)\times (k+3)} \end{aligned}$$

15 $$\begin{aligned} a_3(k):&\, \displaystyle&\frac{2}{5} ,\quad \frac{4}{9} ,\quad \frac{14}{33} ,\quad \frac{56}{143} ,\quad \cdots , \quad \frac{(k-1) \times 7}{(k+2)\times (k+4)} \end{aligned}$$

16 $$\begin{aligned} a_4(k):&\, \displaystyle&\frac{8}{35} ,\quad \frac{24}{77} ,\quad \frac{48}{143} ,\quad \cdots ,\quad \frac{k \times (k-2) \times 9}{(k+1)\times (k+3)\times (k+5)} \end{aligned}$$

17 $$\begin{aligned} a_5(k):&\, \displaystyle&\frac{8}{63} ,\quad \frac{8}{39},\quad \frac{16}{65} ,\quad \cdots ,\quad \frac{(k-1)\times (k-3)\times 11}{(k+2)\times (k+4)\times (k+6)} \end{aligned}$$

18 $$\begin{aligned} a_6(k):&\, \displaystyle&\frac{16}{231} ,\quad \frac{64}{495} , \quad \cdots ,\quad \frac{k \times (k-2)\times (k-4)\times 13}{(k+1) \times (k+3)\times (k+5)\times (k+7)} \end{aligned}$$

19 $$\begin{aligned} a_7(k):&\, \displaystyle&\frac{16}{429} ,\quad \frac{192}{2431} , \quad \cdots ,\quad \frac{(k-1) \times (k-3) \times (k-5)\times 15}{(k+2)\times (k+4) \times (k+6)\times (k+8)} \end{aligned}$$

Using Eqs. (12)–(19), we then deduced the parametric dependence of each of the sequences of $a_l(k)$ on *l*. The results determined in this study for each sequence are presented in Table 3 for even and odd values of *k* and *l*.

**Table 3:** Parametric dependence of the coefficients $a_l(k)$ on *k* and *l* for even and odd values of *l*.

$a_l(k)$ :	Sequence for even *k* and *l*	$a_l(k):$	Sequence for odd *k* and *l*
$a_0(k)$:	$\frac{2l+1}{k+l+1}$	$a_1(k)$:	$\frac{2l+1}{k+l+1}$
$a_2(k)$:	$\frac{k(2l+1)}{(k+l-1)(k+l+1)}$	$a_3(k)$:	$\frac{(k-1)(2l+1)}{(k+l-1)(k+l+1)}$
$a_4(k)$:	$\frac{k(k-2)(2l+1)}{(k+l-3)(k+l-1)(k+l+1)}$	$a_5(k)$:	$\frac{(k-1)(k-3)(2l+1)}{(k+l-3)(k+l-1)(k+l+1)}$
$a_6(k)$:	$\frac{k(k-2)(k-4)(2l+1)}{(k+l-5)(k+l-3)(k+l-1)(k+l+1)}$	$a_7(k)$:	$\frac{(k-1)(k-3)(k-5)(2l+1)}{(k+l-5)(k+l-3)(k+l-1)(k+l+1)}$

Based on observations of the patterns of $a_l(k)$ predicted in Table 3, we have derived the generalized pattern

20 $$\begin{aligned} \displaystyle a_l(k) = \frac{k!!}{(k-l)!!}\, \frac{(k-1)!!}{(k+l+1)!!}\,(2l+1) = \frac{k!}{(k-l)!!\,(k+l+1)!!}\,(2l+1) \end{aligned}$$

for any arbitrary values of *k* and *l*. The derived expansion coefficients’ algorithm $a_l(k)$ given by Eq. (20), independently proved in this study, is established to be equivalent to the expansion coefficients for $x^k$ cited in Eq. (15) in Mathworld^6^.

As a standard application, we consider the multipole series expansion of the plane wave, ${\exp {[i(\mathbf {q} \cdot \mathbf {r}\,)]}}$, and the Coulomb repulsion interaction, ${ \frac{1}{\mid \mathbf {r}_i-\mathbf {r}_j \mid } }$, on the same footing. In both cases, $x^k$ is present in the Taylor expansion of the functions. The multipole series expansion of the plane wave can be expressed as

21 $$\begin{aligned} \displaystyle \exp {[i(\mathbf {q} \cdot \mathbf {r}\,)]} = \sum _{k=0}^{\infty } \frac{(iq \,r)^k\,x^k}{k!} =\sum _{l=0}^{\infty } \sum _{k=l, l+2, \cdots }^{\infty } a_l(k)\,\frac{(iq \, r)^k}{k!}P_l(x) =4\,\pi \sum _{l=0}^{\infty } \sum _{m=-l}^{l} i^l\,j_l(q\,r)\, Y_l^{m*}(\hat{q})\,Y_l^{m}(\hat{r}), \end{aligned}$$

where $\displaystyle Y^m_\ell$ are the standard spherical harmonics, the superscript $^*$ denotes complex conjugation, and

22 $$\begin{aligned} \displaystyle j_l(q\,r) = \sum _{k=l, l+2, \cdots }^{\infty } i^{k-l} \frac{a_l(k)}{(2l+1)\,k!}(q\,r)^k = \sum _{k=l, l+2, \cdots }^{\infty } i^{k-l} \frac{(q \,r)^k}{(k-l)!!\,(k+l+1)!!} \end{aligned}$$

are the regular spherical Bessel functions with the coefficients $a_l(k)$ replaced as defined in Eq. (20). Likewise, the alternative multipole series expansion of the Coulomb repulsion term is given by

23 $$\begin{aligned} \displaystyle \frac{1}{\mid \mathbf {r}_i-\mathbf {r}_j \mid }= & {} (r_i^2 -2r_i\,r_j\,x +r_j^2)^{-1/2} = \sum _{k=0}^{\infty } \left( {\begin{matrix} -\frac{1}{2}\\ k \end{matrix}} \right) (r_i^2+r_j^2)^{-\frac{1}{2}-k}\, (-2r_ir_j)^k\, x^k \nonumber \\= & {} \frac{1}{\sqrt{r_i^2+r_j^2}}\sum _{k=0}^{\infty } \left( {\begin{matrix} -\frac{1}{2}\\ k \end{matrix}} \right) \left( \frac{ - 2r_ir_j}{r_i^2 +r_j^2} \right) ^k\,x^k = \frac{1}{\sqrt{r_i^2+r_j^2}}\sum _{l=0}^{\infty } \sum _{k=l,l+2,\cdots }^{\infty } \frac{b_l(k)}{k!}\, \left( \frac{r_ir_j}{r_i^2 +r_j^2} \right) ^k\, P_l(x) \nonumber \\= & {} \frac{4 \pi }{\sqrt{r_i^2+r_j^2}}\sum _{l=0}^{\infty } \sum _{m=-l}^{l} \tilde{j}_l(r_i,r_j)\, Y_l^{m*}\left( \hat{r_i}\right) \,Y_l^{m}\left( \hat{r_j}\right) , \end{aligned}$$

where we have used

24 $$\begin{aligned} \displaystyle \left( \begin{matrix} -\frac{1}{2} \\ k \end{matrix} \right) = \frac{(2k-1)!!}{(-2)^k\,k!}, \end{aligned}$$

and considered

25 $$\begin{aligned} \displaystyle \tilde{j}_l(r_i,r_j) = \sum _{k=l,l+2, \cdots }^{\infty } \frac{b_l(k)}{(2l+1)\,k!}\, \left( \frac{r_ir_j}{r_i^2 +r_j^2} \right) ^k = \sum _{k=l,l+2, \cdots }^{\infty } \frac{(2k-1)!!}{(k-l)!!\,(k+l+1)!!}\, \left( \frac{r_ir_j}{r_i^2 +r_j^2} \right) ^k \end{aligned}$$

as a spherical Bessel-like function with the coefficient ${b_l(k)=a_l(k)\,(2k-1)!!}$. One can note the similarity between Eqs. (22) and (25), with the difference seen only in the expansion coefficients. It can also be observed that

26 $$\begin{aligned} \displaystyle \frac{1}{\mid {\mathbf {r}}_i-{\mathbf {r}}_j \mid } \approx \frac{1}{\sqrt{r_i^2+r_j^2}} \end{aligned}$$

if the lowest order approximation ($l=k=0$) is considered.

The conventional multipole expansion series of the Coulomb repulsion term, on the other hand, can be derived by rearranging the terms of the Binomial expansion as

27 $$\begin{aligned} \displaystyle \frac{1}{\mid {\mathbf {r}}_i-\mathbf {r}_j \mid }= & {} \left( r_i^2 -2r_i\,r_j\,x +r_j^2\right) ^{-1/2} = \frac{1}{r_>} \left( 1 -2tx +t^2\right) ^{-1/2} =\frac{1}{r_>}\sum _{k=0}^{\infty } \left( {\begin{matrix} -\frac{1}{2}\\ k \end{matrix}} \right) \left( t^2-2tx\right) ^k \nonumber \\= & {} \frac{1}{r_>}\sum _{k=0}^{\infty } \sum _{\lambda = 0}^k \left( {\begin{matrix} -\frac{1}{2}\\ k \end{matrix}} \right) \left( {\begin{matrix} k\\ \lambda \end{matrix}} \right) \left( t^2\right) ^{k-\lambda }(-2tx)^{\lambda } =\frac{1}{r_>}\sum _{k=0}^{\infty } \sum _{\lambda = 0}^k \frac{n!}{(n-k)! \lambda !}(-2)^{\lambda }\, t^{2k-\lambda }\,x^{\lambda }, \end{aligned}$$

where $${t=r_</r_>}$$, $${r_<=\mathrm {min }(r_i, r_j)}$$, $${r_>=\mathrm {max }(r_i, r_j)}$$, and $${n=-1/2}$$.

With the substitution of the series expansion of $x^k$ as defined in Eq. (3), and further evaluation of the combinatorics using Eq. (24), the summation in Eq. (27) simplifies to

28 $$\begin{aligned} \displaystyle \frac{1}{\mid \mathbf {r}_i-\mathbf {r}_j \mid } = \frac{1}{r_>}\sum _{l=0}^{\infty } h_l(t)\,P_l(x), \end{aligned}$$

where

29 $$\begin{aligned} \displaystyle h_l(t)= & {} \sum _{\lambda \ge l, l+2, \cdots }^{\infty } \frac{(-2)^{\lambda }a_l(\lambda )}{\lambda !} \sum _{k \ge \lambda ,\lambda +1, \cdots }^{\infty } \frac{(2k-1)!!}{(-2)^k\,(k-\lambda )!}t^{2k-\lambda }\nonumber \\= & {} \sum _{\lambda \ge l, l+2, \cdots }^{\infty } \frac{(-2)^{\lambda }(2\lambda +1)}{(\lambda -l)!!\; (\lambda +l +1)!!} \sum _{k \ge \lambda ,\lambda +1, \cdots }^{\infty } \frac{(2k-1)!!}{(-2)^k\,(k-\lambda )!}t^{2k-\lambda }. \end{aligned}$$

According to Eqs. (28) and  (29), the conventional multipole expansion of the Coulomb repulsion term ^7–9^,

30 $$\begin{aligned} \displaystyle \frac{1}{\mid \mathbf {r}_i-\mathbf {r}_j \mid } = \sum _{l=0}^{\infty } \frac{r_<^l}{r_>^{l+1}}\,P_l(x), \end{aligned}$$

arises from a special case where ${l=\lambda =k}$. This therefore implies that the completeness of the conventional multipole expansion of the Coulomb repulsion term can only be verified if the higher order terms (${\lambda > l}$ and ${k \ge \lambda }$) of the expansion series are shown to be vanishing. In verifying the completeness of the conventional multipole expansion of the Coulomb repulsion term, we expand Eq. (29) further to obtain

31 $$\begin{aligned} \displaystyle h_l(t)= & {} \sum _{\lambda \ge l, l+2, \cdots } ^{\infty } \frac{(2\lambda -1)!!(2\lambda +1)\,t^{\lambda }}{(\lambda -l)!!\; (\lambda +l +1)!!} \nonumber \\&\times \;\; \sum _{k=0}^{\infty } 1 - \frac{(2\lambda +1)}{1!}\left( \frac{t^2}{2}\right) -\frac{(2\lambda +3)(2\lambda +1)}{2!}\left( \frac{t^2}{2}\right) ^2 + \frac{(2\lambda +5)(2\lambda +3)(2\lambda +1)}{3!}\left( \frac{t^2}{2}\right) ^3 \cdots \nonumber \\= & {} \sum _{\lambda \ge l, l+2, \cdots }^{\infty } \frac{(2\lambda -1)!!(2\lambda +1)\,t^{\lambda }}{(\lambda -l)!!\; (\lambda +l +1)!!\, (2 \lambda !!)} \times \sum _{k=0}^{\infty } (-1)^k \, \frac{(2 \lambda +k)!!)}{k!}\left( \frac{t^2}{2}\right) ^k . \end{aligned}$$

It can be shown by Taylor expansion that

32 $$\begin{aligned} \displaystyle \frac{1}{\left[ 1+t^2\right] ^{\lambda + \frac{1}{2}}} = \frac{1}{(2 \lambda !!)} \sum _{k=0}^{\infty } (-1)^k \, \frac{(2 \lambda +k)!!)}{k!}\left( \frac{t^2}{2}\right) ^k . \end{aligned}$$

Substituting Eq. (32) into Eq. (31), we obtain the function

33 $$\begin{aligned} \displaystyle h_l(t) =\sum _{\lambda \ge l, l+2, \cdots }^{\infty } \frac{(2\lambda -1)!!(2\lambda +1)}{(\lambda -l)!!\; (\lambda +l +1)!!}\; \frac{t^{\lambda }}{\left[ 1+t^2\right] ^{\lambda + \frac{1}{2}}} \end{aligned}$$

as a summation of a single index just as the related function presented in Eq. (25). In the conventional form, the analytical function given by Eq. (32) is approximated to unity and the series in Eq. (33) is truncated by considering the special case of ${\lambda = l}$ only. The approximation of the analytical function and the truncation of the expansion leads to inaccuracy and incompleteness of the conventional multipole expansion of the Coulomb repulsion term.

## Conclusion

Our goal in this paper is to expand ${\cos ^k \theta }$ in the basis of the Legendre polynomials and consequently use it for the multipole expansion of the plane wave as well as the electron-electron repulsion term. The multipole expansion method, as a technique for separating a 3D function into a product of radial and angular components, is not unique. However, its application to the standard Coulomb interaction term in this paper leads to two unique but equivalent multipole expansion series of the electron-electron repulsion term. We show that the conventional multipole expansion of the Coulomb repulsion term is a special case in which a three indices’ expansion series in the Legendre polynomial basis is truncated to a single-index summation. The alternative multipole expansion series of the Coulomb repulsion term, as defined in Eqs. (23) and (25), is a two-indices expansion. These unique multipole expansion series of the Coulomb repulsion term, to the best of our knowledge, have not been reported in literature. The application of the derived alternative multipole expansion of the Coulomb repulsion term in solving the electron correlation problem in electronic structure theory is a subject of our current research interest.
